# How to quantify conduits in wood?

**DOI:** 10.3389/fpls.2013.00056

**Published:** 2013-03-18

**Authors:** Alexander Scholz, Matthias Klepsch, Zohreh Karimi, Steven Jansen

**Affiliations:** ^1^Institute for Systematic Botany and Ecology, Ulm UniversityUlm, Germany; ^2^Golestan UniversityGorgan, Iran

**Keywords:** bordered pit, pit membrane, tracheid, vessel, vessel element, wood anatomy

## Abstract

Vessels and tracheids represent the most important xylem cells with respect to long distance water transport in plants. Wood anatomical studies frequently provide several quantitative details of these cells, such as vessel diameter, vessel density, vessel element length, and tracheid length, while important information on the three dimensional structure of the hydraulic network is not considered. This paper aims to provide an overview of various techniques, although there is no standard protocol to quantify conduits due to high anatomical variation and a wide range of techniques available. Despite recent progress in image analysis programs and automated methods for measuring cell dimensions, density, and spatial distribution, various characters remain time-consuming and tedious. Quantification of vessels and tracheids is not only important to better understand functional adaptations of tracheary elements to environment parameters, but will also be essential for linking wood anatomy with other fields such as wood development, xylem physiology, palaeobotany, and dendrochronology.

## Introduction

Vessel elements and tracheids play a crucial role in the transport of water from roots to leaves. Both cell types, also called tracheary elements or simply conduits, show a wide anatomical diversity with respect to their size, shape, arrangement, and grouping (Carlquist, 2001). Fiber-tracheids and libriform fibers are interpreted as non-water conducting cells (Sano et al., 2011). Tracheary elements have been studied by plant anatomists for many years and provide valuable information to a wide range of wood related study fields, ranging from wood identification and palaeobotany to plant ecology and physiology (Carlquist, 2001; Tyree and Zimmermann, 2002; Fonti et al., 2010; Pittermann, 2010; Gasson, 2011; Choat et al., 2012).

Traditionally, vessels and tracheids have been characterized using light microscopy by measuring conduit diameter, vessel element and tracheid length, and vessel density. While these measurements provide useful characters based on relatively simple techniques, a large number of characteristics quantifying the three dimensional (3D) network of vessels and/or tracheids remains unexplored. While some 3D characteristics can be time-consuming and not straightforward to students interested in xylem anatomy, others can be measured using relatively simple standard techniques. Furthermore, definitions and methodology of anatomical features may vary considerably among anatomists, illustrating the difficulty in obtaining unbiased and reliable data.

Only few textbooks on wood anatomy include precise and clear instructions on technical details of conduits (e.g., Chaffey, 2002), and the wide range of methods applied to measure vessels, vessel elements, and tracheids is found in a large number of diverse papers. While there are various techniques available for quantifying xylem conduits, each one has its own advantages and drawbacks. Conduit parameters may not only provide additional structural information, but also valuable insight into hydraulic functionality and ecological traits. Because water conducting xylem cells are extremely variable, a method that may work perfectly well for diffuse-porous angiosperms may not be applicable to ring-porous woods. Moreover, collection of various samples and sufficient repetition is frequently required in order to deal with intra-tree, intraspecific, and interspecific variation (Schweingruber et al., 2006; Fichtler and Worbes, 2012).

This paper intends to give an overview of some techniques that can be applied to determine quantitative characteristics of vessels and tracheids. It is not our purpose to summarize all available methods, but to present a selection of conduit characters together with applicable and established measuring approaches, potential problems and shortcomings, as well as references to further literature. We hope such review will encourage students to be creative in modifying existing protocols, or in developing new techniques. We also welcome the online availability of anatomical protocols such as the recently established Prometheus website (Sack et al., 2010; Nicotra and McIntosh, 2011).

## Conduit diameter

A summary of the directly measurable conduit characters and derived characters are listed in Tables 1 and 2. The conduit diameter is one of the most important parameters since it directly affects hydraulic conductivity (*K*_h_). Based on the Hagen–Poiseuille law the diameter scales to the fourth power of the conductance (Giordano et al., 1978; Ewers and Fisher, 1989b; Ewers et al., 1990). High hydraulic efficiency (or low hydraulic resistance) is associated with high stomatal conductance and thus indirectly with the photosynthetic capacity of a plant (Brodribb and Feild, 2000).

**Table 1:** **Overview of quantitative, directly measurable conduit characters with reference to their acronym, definition, measurement procedure, microscope technique, and units**.

**Acronyms**	**Definition**	**Measurement procedure**	**Technique**	**Units**
*A*_PA_	Pit aperture surface area	Min. 50 measurements of pits from different vessels	SEM	μm^2^
*A*_PIT_	Intervessel pit surface area = area occupied by the pit border or the intervessel pit membrane	Min. 50 measurements of pits from different vessels	SEM	μm^2^
*D*	Arithmic vessel diameter = the simple average of the equivalent circle diameters	Min. 100 vessels; distinguish earlywood from latewood	LM	μm
*D*_F_	Fiber lumen diameter = arithmic diameter corresponding to equivalent circle diameter of fiber lumina	Min. 100 fibers; distinguish earlywood from latewood	LM	μm
*D*_H_	Hydraulic diameter	Min. 100 vessels; distinguish earlywood from latewood	LM	μm
	1. Σ D^5^/ΣD^4^			
	2. (ΣD^4^/N)^1/4^			
*D*_MAX_	Maximum vessel diameter	Min. 100 vessels; distinguish earlywood from latewood	LM	μm
*D*_PA_	Diameter of outer pit aperture as measured at the widest part of the opening	Min. 50 pits	SEM	μm
*D*_PC_	Pit chamber depth = distance from the pit membrane to the inner pit aperture	Min. 25 pits	TEM	μm
*D*_PM_	Horizontal pit membrane diameter at its widest point = horizontal pit border diameter = pit size	Min. 50 pits	LM or SEM	μm
*D*_RL_	Vessel diameter corresponding to mean vessel lumen resistivity	Min. 100 vessels; distinguish earlywood from latewood	LM	μm
*D*_T_	Tangential vessel diameter	Min. 100 vessels; distinguish earlywood from latewood	LM	μm
*F*_C_	Intervessel contact fraction = portion of vessel wall in contact with other vessels as based on transverse sections	Min. 100 vessels; distinguish earlywood from latewood	LM	–
*F*_PF_	Pit-field fraction = ratio of intervessel surface area occupied by intervessel pits to total intervessel wall area	Min. 5 intervessel walls as viewed in tangential longitudinal sections	SEM or LM	–
*F*_VM_	Vessel multiple fraction = ratio of grouped vessels to total number of vessels	Min. 50 vessel groups	LM	–
*L*_vw_	Intervessel wall length = length of vessel wall in contact with other vessels as based on transverse sections	Min. 100 vessels; distinguish earlywood from latewood	LM	μm
*L*_F_	Fiber length	Min. 50 fibers	LM	μm
*L*_MAX_	Maximum vessel length	Air injection, stem-shorting or silicon injection of min. 3 samples	-	cm
*L*_T_	Tracheid length	Min. 50 tracheids	LM	μm
*L*_V_	Vessel length as based on vessel length distribution data	Silicon injection of min. 3 samples	LM	cm
*L*_VE_	Vessel element length	Min. 50 vessel elements	LM	μm
*T*_FW_	Fiber wall thickness = total wall thickness measured as the double wall between 2 adjacent fibers	50 measurements	LM	μm
*T*_PM_	Intervessel pit membrane thickness measured at its thickest point	Min. 25 measurements	TEM	nm
*T*_VW_	Intervessel wall thickness measured as the double intervessel wall in the middle of adjacent vessels	Min. 50 measurements	LM	μm
*VA*	Average vessel area	Min. 100 measurements	LM	μm^2^
*V*_D_	Vessel density = number of vessels per mm^2^	Min. 5 measurements	LM	mm^2^
*V*_G_	Vessel grouping index = ratio of total number of vessels to total number of vessel groupings (incl. solitary and grouped vessels)	Min. 50 vessel groups	LM	–
*V*_S_	Solitary vessel index = ratio of solitary vessels to total vessel groupings (incl. solitary and grouped vessels)	Min. 50 vessel groups	LM	–

The number of measurements given under measurement procedure is a recommendation and depends on the anatomical variation within a given sample and species. LM, light microscopy; SEM, scanning electron microscopy; TEM, transmission electron microscopy; –, no units.

**Table 2:** **Overview of derived quantitative conduit characters with reference to their acronym, definition, formula, and units**.

**Acronyms**	**Definition**	**Formula**	**Units**
*A*_P_	Total intervessel pit membrane surface area per vessel area	*A*_V_ × *F*_P_	mm^2^
*A*_V_	Vessel surface area	Π × *D*_RL_ × *L*_V_	mm^2^
*F*	Vessel lumen fraction (*NF* = non-vessel lumen fraction)	*F* = *V*_D_ × *V*_A_	–
		NF = 1 – *F*	
*F*_LC_	Vessel contact length fraction	*L*_C_/*L*_V_ = 1 – *V*_S_	–
*F*_P_	Pit fraction = mean fraction of the vessel area occupied by intervessel pits	*F*_C_ × *F*_PF_	–
*L*_C_	Total inter vessel contact length = average contact length between adjacent vessels = average length of vessel end walls	*L*_V_ × (1 – *V*_S_)	cm
*MI*	Mesomorphy index following Carlquist (1977)	VI × *L*_VE_	μm^2^ mm^−2^
*VI*	Vulnerability index following Carlquist (1977)	D/*V*_D_	μm mm^−2^
(*T*_VW_/*D*_MAX_)^2^	Theoretical vessel implosion resistance	(*T*_VW_/*D*_MAX_)^2^	–

Also, vulnerability to freezing-induced cavitation is directly associated with conduit diameter: freeze-thaw events are experimentally demonstrated to affect plants with a conduit diameter above 30 μm (Davis et al., 1999). There is wood anatomical evidence that species with wide tracheary elements are more vulnerable to drought-induced cavitation than those with narrow conduits (Pockman and Sperry, 2000; Carlquist, 2001; Christman et al., 2012).

Several definitions of conduit diameters can be distinguished, although the difference between these definitions does not tend to be very pronounced. The smallest value is usually obtained for the arithmetic circle diameter (*D*), followed by the mean lumen resistivity diameter (*D*_RL_), the tangential diameter (*D*_T_), and the hydraulic diameter (*D*_H_). Further explanation and calculation of the diameters is discussed below.

Because conduit diameters frequently differ along a gradient from the most recently developed wood toward the pith, the exact location of the wood sample or the section within the stem should be carefully considered. In ring-porous species, early and late wood should be treated separately. It is recommended that a minimum of 50 vessels is measured per sample, although the number of sectors and vessels depends on variation of conduit size and density.

### Tangential vessel diameter (*D*_T_) [μm]

This is the standard way of measuring the conduit diameter for wood identification and wood anatomical descriptions. The tangential vessel diameter is measured along its widest tangential axis for a minimum of 50 randomly selected vessels, and the minimum, maximum (*D*_MAX_), and average values are calculated. While the approach is straightforward and fast, this method does not consider the conduit shape and the hydraulic conductance of conduits.

### The equivalent circle diameter (*D*) [μm]

**Figure 1:**
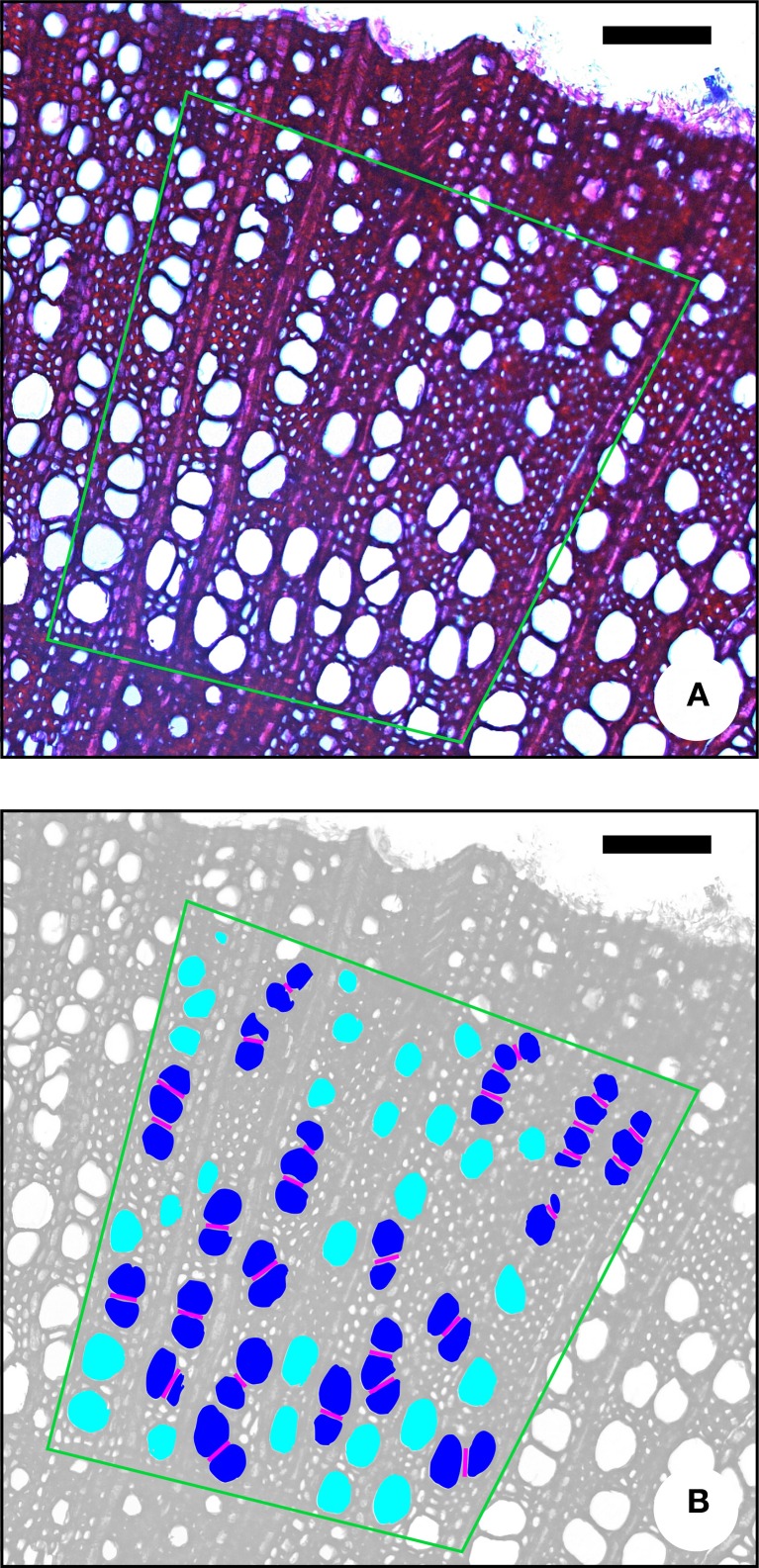
**Illustration of some characters that can be measured on a transverse section of *Prunus domestica*. (A)** The original image, **(B)** the same image modified for image-analysis. Diameter **(D)** and perimeter **(P)** are easily measured on transverse sections. For calculating the vessel grouping index (*V*_G_), the number of vessel groups and the total number of vessels has to be determined. Light blue, solitary vessels (one vessel per group); dark blue, vessel multiples (two or more vessels per group). Pink lines, (inter) vessel contact length (*L*_VW_). Green polygon, AOI (area of interest), covering earlywood and latewood. All vessels outside the AOI were excluded. Scale bar = 100 μm.

The equivalent circle diameter (*D*) is the diameter of the circle having the same area as the measured cell (Figure 1, Equation 1). It is also possible to calculate *D* by taking the perimeter of each individual conduit (Equation 2). Both calculations lead to slightly different results. Since conduits are frequently elliptical and rarely perfect circles as seen in transverse sections, it is sometimes more accurate to calculate the arithmetic diameter instead of the tangential vessel diameter (*D*_T_). A similar approach can be applied for conduits with a square shape (e.g., gymnosperm tracheids), or even other geometrical forms (Lewis, 1992; Sperry and Sullivan, 1992; Sperry et al., 1994; Davis et al., 1999).

(1) $$D=\frac{P}{\pi }$$

Calculation of *D* using the conduit perimeter (*P*)

(2) $$D=\sqrt{\frac{4A}{\pi }}$$

Calculation of *D* using the conduit surface area (*A*)

### The hydraulic diameter (*D*_H_) [μm]

The hydraulic diameter (or hydraulically weighted diameter) is based on the equivalent circle diameter *D* and has been introduced to reflect the actual conductance of conduits. Based on the Hagen–Poiseuille law (Equation 3), a few large conduits may transport an equal amount of water as many small ones (Tyree and Zimmermann, 2002).

(3) $$K_{\text{h}}=\frac{\pi D^{4}}{128\eta }\;R_{\text{L}}=\frac{128\eta }{\pi D^{4}}$$

Hydraulic conductivity (*K*_h_) and lumen resistivity (*R*_L_) based on the Hagen–Poiseuille law.

*D* is the diameter and η is the viscosity index of water (1.002 × 10^−9^ MPa s at 20°C). *K*_h_ is the hydraulic conductivity [m^4^/MPa^−1^ × s^−1^] and *R*_L_ is the m^−4^ MPa s lumen resistivity [MPa × s/m^−4^].

Most conduits, however, do not behave like ideal capillaries because of additional resistance offered by an irregular conduit shape, axial changes in conduit diameter, and wall sculpturing such as warts, vestures, helical thickenings, and perforation plates (Akachuku, 1987; Martre, 2000; Sperry et al., 2005; Hargrave et al., 2006; Christman and Sperry, 2010).

There are two main approaches for calculating *D*_H_, which have both been widely applied.

(4) $$D_{H}={\left(\frac{\sum D^{4}}{N}\right)}^{\text{​​}\frac{1}{4}}$$

Calculation of *D*_H_ after Tyree and Zimmermann (2002).

(5) $$D_{H}=\frac{\sum D^{5}}{\sum D^{4}}$$

Calculation of *D*_H_ after Sperry et al. (1994).

*D*_H_ calculated following Equation 4 represents the mean diameter that all of the vessels in a stem would have in order to correspond to the overall conductivity for the same numbers of conduits (*N*), while *D*_H_ following Equation 5 is a statistic that simply weights the conduit size, and which may not be recommended for ring-porous species.

### Lumen resistivity diameter (*D*_RL_) [μm]

*D*_RL_ is the conduit diameter based on lumen resistivity (*R*_L_). It is calculated by applying the Hagen–Poiseuille equation of each conduit lumen diameter based on its perimeter or surface area. The individual conduit resistivities are then summed up to obtain the total resistivity. Several sectors of a stem are measured to obtain an average. The *R*_L_ of an average vessel is calculated by dividing the sum of the lumen resistivites by the number of vessels measured. This value is then put back into the Hagen–Poiseuille Equation (3) to get *D*_RL_ (Gibson et al., 1985; Ewers and Fisher, 1989a; Tyree and Zimmermann, 2002; Sperry et al., 2005; Hacke et al., 2006; Pittermann and Sperry, 2006; Hacke et al., 2007).

### Theoretical vessel implosion resistance (*T*_VW_/*D*_MAX_)^2^

This mechanical parameter is calculated based on the double intervessel wall thickness (*T*_VW_) divided by the maximum diameter of the vessel (*D*_MAX_). In brief, the tension of the water column creates a force that acts on the conduit cell walls, pulling these toward the center of the conduit. If the tension increases due to drought stress, conduit walls could theoretically implode, although such observations have only been observed in xylem of leaves and not in stems. For more information we recommend Hacke et al. (2001) and Pittermann et al. (2006).

## Conduit length

The length of vessel elements and tracheids, which reflect the length of fusiform cambium initial cells, have been used as a major criterion in establishing evolutionary traits in wood anatomy (Bailey and Tupper, 1918). From a functional point of view, however, the total vessel length plays a more important role in determining hydraulic resistance than the vessel element length, although the vessel length remains little studied despite the development of various techniques (Zimmermann and Jeje, 1981; Cohen et al., 2003). Vessel element length has been suggested to be a sensitive character for xeromorphy or mesomorphy (Carlquist, 1977), but functional interpretations remain difficult and lack experimental evidence.

### Tracheid length (*L*_T_) [μm] and vessel element length (*L*_VE_) [μm]

Maceration of wood slivers is a common technique applied to determine tracheid length and vessel element length. Good results can be obtained using Franklin's solution (Franklin, 1945), although there are various alternative techniques. Care should be taken not to measure incomplete or broken cells.

An alternative method to measure vessel element length is the microcasting technique based on André (2005), which is more time consuming, and not appropriate for quantifying tracheid length. In summary, air-dried samples are immersed in a liquid of a two component silicon mixture and put progressively under vacuum for 20–30 min. The samples are then placed in a freezer overnight to avoid fast polymerization of the mixture and to let the silicon slowly fill the degassed vessels. Once the silicon has become hard after drying at room temperature or in an oven, the cell walls are destroyed by immersing the samples first in Franklin's solution for 1 night at 60°C, and then into 150 ml 72% (weight percentage) sulphuric acid hydrate (H_2_SO_4_). It is important to change the sulphuric solution repeatedly until the solution does no longer turn very dark to brown. Twenty four hours in H_2_SO_4_ is generally sufficient. The casts are then neutralized by 300 ml of a sodium bicarbonate solution. Further treatment with 50% Parazone or sodium hypochlorite will make the casts clear and white in color. After washing in water, the microcasts can be studied using light microscopy using water or glycerol as mounting medium. For further details on this method see André (2005).

Another method for determining tracheid length (*L*_T_), which does not require macerated tissue, but longitudinal sections, has been suggested for tracheid length measurements in gymnosperm wood (Ladell, 1959; Wilkins and Bamber, 1983; Baas et al., 1986). Briefly, the number of tracheids (*N*_T_) between two radial lines with a known distance (Δ*LL*) is divided by the number of tracheids ending (*N*_TTips_) between these lines (Equation 6). This method assumes random distribution of tracheids and has been used far less frequently than macerations, although similar results were obtained when comparing both methods (Wilkins and Bamber, 1983; Baas et al., 1986). Ladell's method allows precise location of the measured cells, but identification of tracheid tips could be difficult for narrow (<20 μm) tracheids. Also, this method is not applicable for measuring vessel elements.

(6) $$L_{\text{T}}=\frac{N_{\text{T}}\times \Delta LL}{N_{\text{TTips}}}$$

Calculating tracheid length according to Ladell (1959).

### Vessel length (*L*_V_) [cm]

The following techniques have been suggested to calculate the average vessel length and vessel length distributions: the pigment injection (Greenidge, 1952), air-injection (Skene and Balodis, 1968; Zimmermann and Jeje, 1981; Cohen et al., 2003), cinematographic method (Bosshard and Kucera, 1973; Huggett and Tomlinson, 2010), paint injection (Zimmermann and Jeje, 1981; Ewers and Fisher, 1989a), wire insertion (Kanai et al., 1996), X-ray distribution of titanium mixed with a paint solution (Middleton, 1989), silicon injection (Sperry et al., 2005), and high resolution computed tomography (Brodersen et al., 2011).

The silicon injection method, which was inspired by the microcasting technique (André, 2005; see above), is probably the most widely applied method nowadays and may provide accurate data if sufficient samples are analysed (Wheeler et al., 2005; Hacke et al., 2007). The air-injection method has been frequently applied to estimate the maximum vessel length (Greenidge, 1952; Cohen et al., 2003). However, comparison of the maximum vessel length based on silicon and air-injection suggests that the air-injection may overestimate the maximum vessel by nearly 50% or even more. Nevertheless, if the aim is to determine the length of a stem segment that has no open vessels, the air-injection method is a fast method.

Air-injection can be applied by connecting the distal end of a branch to a syringe or a pressure tank. Embolism removal by flushing is highly recommended for avoiding possible overestimations of the maximum *L*_V_ values, because air may pass rather easily through the pores of intervessel pit membranes in embolized vessels in which the air-water interfaces would be broken. Therefore, flushing samples before determining the maximum *L*_V_ or the vessel length distribution by air-injection is highly advisable to reestablish the intervessel air-water interfaces. The proximal end can be plunged into a water bath to which a detergent is added or by applying a soap solution or heavy lubricating oil to the cut surface (Greenidge, 1952). In general, a pressure of ca. 100 kPa is applied to test if air-bubbles emerge from the cut end. The stem is cut under water from the proximal end until at least one open vessel, which corresponds to the maximum vessel length, is reached. A hand lens can be used to detect the air-bubbles; only a regular stream of bubbles is indicative of an open vessel. The air-injection method could be problematic for species with a large (sometimes hollow) pith structure through which air can travel easily (e.g., bamboo species). In that case, the pith can be blocked off with superglue or any other sealing solution.

A relatively simple test to find out whether maximum vessel length values obtained are reliable is to conduct a stem-shortening experiment. Plotting the mean increase of the flow rate after successive stem shortening against the segment length will show a distinct increase of the flow rate when one or two vessels are cut open. The explanation for this increase is that intervessel pits generally account for >50% of the total hydraulic resistance (Choat et al., 2008). In general, the combination of air-injection, silicon injection, and stem-shortening flow measurements is recommended for detecting possible errors or discrepancies.

Although fresh material is recommended for applying the silicon injection technique, more or less similar results can be obtained with dried material after rehydration and degassing overnight using a vacuum pump (personal observations). In general, five replicates per species should be measured to obtain an accurate estimation of the vessel length distribution. Only a small amount of silicon is needed, e.g., a mixture of 11 ml would be sufficient for 5–10 stems, depending on their vessel diameter and length. The Rhodorsil RTV-141 A and its corresponding hardening component Rhodorsil RTV-141 B are recommended, although other 2-compound silicone solutions with similar pot life and polymerization properties could be tried, e.g., Sorta-Clear, Q-Sil 218, OPT Opti-tec. It is important, however, to test whether or not the silicon is able to pass through intervessel pit membranes. In order to distinguish injected vessels from non-filled ones in transverse sections, a fluorescent dye is added to the silicon solution, for instance Uvitex OB. Other pigments have also been used to color silicon, but caution is needed to make sure that these are not too large and potentially clog up the vessel lumina.

After degassing, the silicon mixture is injected with a pressure of 0.3–0.5 MPa for 3–4 h, or sometimes even overnight. The pressure and duration could vary between species and should therefore be adjusted for each case in particular. The use of a Scholander pressure bomb or a pressure collar can be used for this purpose. We frequently use four double-ended pressure chambers that are connected via a manifold to inject four samples simultaneously. One side of the double-ended pressure chamber is closed with a rubber gasket, while a stem branch is inserted through the other opening. Samples can be injected in an acropetal or basipetal direction without differences in vessel length distribution. However, it is known that spatial distribution patterns of vessels near nodes, side branches, and petioles of leaves and flowers can be significantly different from an otherwise random vessel distribution (Salleo et al., 1984; André et al., 1999; Rancić et al., 2010). In general, we recommend injecting basipetally, selecting an injection point that avoids side branches, nodes, and leaves as much as possible. In this way, a random distribution of vessel ends would be more likely and would result in limited impact on the vessel length distribution.

Samples can be left to dry at room temperature for 5–6 h, which is long enough to let the silicon harden, but too short for the stem to desiccate completely. Generally, the Rhodorsil A/B mixture hardens after 5 h at 25°C. It is also possible to speed up the drying by putting samples in the oven, although this has the disadvantage that the samples will become dried out completely, which usually makes sectioning more difficult. The samples can then be cut to measure the amount of vessels filled with silicon at different lengths from the injection point. Because vessels are extremely short-skewed, most vessels will end at a short distance from the injection point. Therefore, it is very important to make more sections close to the injection point (Zimmermann and Potter, 1982; Tyree and Zimmermann, 2002). Equation 7 can be used to calculate the sectioning distances for a particular number of sections (*n*):

(7) $$L_{i}={\left(L_{\text{MAX}}/L_{\text{MIN}}\right)}^{\left[(i\;-\;1)/(n\;-\;1)\right]}$$

Calculation of the sectioning distances.

At first, transverse sections are cut, starting from the distal end of the injection point, to detect the distance where the number of filled vessels exceeds 2% (*L*_MAX_, *i* = *n*). Then, a section is cut at 0.6 cm distance (*L*_MIN_ = 0.6, *i* = 1) from the injection point, with additional sections between *L*_MIN_ and *L*_MAX_ calculated using Equation 7.

The Uvitex-stained silicon can easily be detected using fluorescent light microscopy, at 387 nm, after cutting sections with a sliding microtome. For embedding the sections, a mounting medium should be selected that does not remove the fluorescent character of the silicon. We therefore recommend mounting sections in glycerol.

Vessel length distributions are analysed from a radial sector in the most recently developed growth ring. Two approaches can be applied, depending on axial changes in the vessel density. If vessel density is constant across the stem length, the total vessel number of filled and non-filled vessels can be counted at 0.6 cm from the injection point only, and only filled vessels have to be counted in consecutive sections. If the vessel density differs along the stem axis, filled and non-filled vessels have to be counted in all sections. For diffuse-porous species, a complete radial sector is analysed with an image analysis program. The number of images per sample and the size of the growth ring area depend on the vessel size and vessel density, and should be adapted accordingly. The vessel length distribution and the average vessel length (*L*_V_) can then be calculated for each replicate on the basis of equations reported by Wheeler et al. (2005) and Sperry et al. (2005). Excel spreadsheets that can be used to calculate vessel length parameters are also available from John Sperry's website (http://biologylabs.utah.edu/sperry/methods.html). Additional information and pictures illustrating vessel length measurements have also been posted on Anna Jacobsen's website (http://www.csub.edu/~ajacobsen/Vessel%20Length%20Methods.pdf).

## Vessel arrangement and connectivity

While gymnosperms and vesselless angiosperms show a relatively uniform distribution of tracheids, vessels in eudicots can be arranged in various patterns, varying from mainly solitary to highly connected vessels. Traditionally, rather limited attention has been paid to the spatial distribution of vessels and their possible role in the hydraulic system of plants. Earlier work based on the connectivity between vessels has illustrated that vessels are usually randomly arranged (Zimmermann and Tomlinson, 1966; Braun, 1970) except near nodes and in leaf and flower abscission zones (Salleo et al., 1984; André et al., 1999; Rancić et al., 2010). Furthermore, vessels nearly always start and end in connection to another vessel, which means that vessels do not end or start blindly (Zimmermann and Tomlinson, 1966; Braun, 1970). The axial distribution and connectivity of vascular bundles in monocots remains studied in only few taxa.

More recently, novel attention to intervessel connectivity has increased because of ecological differences in vessel arrangement between closely related species (Carlquist, 2009; Lens et al., 2011), the rare-pit hypothesis (see also *A*_P_, interconduit pit membrane area; Wheeler et al., 2005; Lens et al., 2011), ion-induced increase of hydraulic conductivity (Jansen et al., 2011; Nardini et al., 2012), and the importance of vessel connectivity for a recently developed model on hydraulic efficiency and safety (Loepfe et al., 2007; Martínez-Vilalta et al., 2012). As a result, the three dimensional distribution of xylem vessels has received attention at various scales, using different terms for related concepts, such as vessel grouping, aggregation, connectivity, redundancy, sectoriality, integration, and segmentation (Martínez-Vilalta et al., 2012).

### Vessel density (*V*_D_) [mm^−2^]

In general, vessel density is quantified as the average number of conduits per 1 mm^2^. The number of measurements should depend on the variation found within a transverse section. Therefore, earlywood and latewood should be considered separately for ring-porous woods. Vessel density is useful to calculate average vessel length (*L*_V_; see above) and can thus be measured in combination with *L*_V_. Measuring the number of tracheids per mm^2^ is not so common, but can be facilitated by using an automated image analysis program.

### Vessel lumen fraction (*F*) [mm^2^ × MM^−2^]

Vessel lumen fraction (*F*) is a good indicator for stem mechanical strength and hydraulic conductivity. The higher the proportion of vessel lumina in a sample is, the lower the support tissue fraction for a given stem diameter (Jacobsen and Ewers, 2005; Preston et al., 2006). According to Equation 8, it is calculated using the mean vessel density (*V*_D_) and average vessel area (*V*_A_) (Zanne et al., 2010; Martínez-Vilalta et al., 2012). Also wood density, another trait determining wood mechanical strength, is directly linked to the non-lumen fraction (*NF*). The denser the wood is, the larger the proportion of the non-lumen fraction (*NF*) (Zanne et al., 2010).

(8) $$F=V_{\text{D}}\times V_{\text{A}}\;NF=1-F$$

Vessel lumen fraction and non-lumen fraction.

### Vessel grouping indices (*V*_G_, *V*_S_, *F*_VM_)

The vessel grouping index *V*_G_ as defined by Carlquist (2001) corresponds to the total number of vessels divided by the total number of vessel groupings. The total number of groups is the sum of solitary vessels plus vessel clusters and radial multiples. A solitary vessel counts as one vessel group. A vessel grouping index of 1 indicates exclusively solitary vessels, the higher the index, the greater the degree of vessel grouping (Figure 1).

This index does not take into account the diameter of vessels. *V*_G_ is the opposite of the vessel multiple fraction (*F*_VM_), which represents the ratio of vessel groupings to the total number of vessels. An alternative parameter for the vessel grouping index has been suggested by Mencuccini et al. (2010) and Martínez-Vilalta et al. (2012), who measured connectivity on transverse sections using a point pattern analysis and a piecewise Geyer model.

The solitary vessel index (*V*_S_) is defined as the ratio of total number of solitary vessels to total number of vessel groupings (including solitary and grouped vessels). It resembles the proportion of vessel length not in contact with adjacent vessels, and is also used to calculate inter vessel contact length (*L*_C_) and vessel contact length fraction (*F*_LC_) (Wheeler et al., 2005).

(9) $$V_{\text{G}}=\frac{N_{\text{vessels}}}{N_{\text{groupings}}}\;V_{\text{S}}=\frac{N_{\text{solitary vessels}}}{N_{\text{groupings}}}\;F_{\text{VM}}=\frac{N_{\text{groupings}}}{N_{\text{vessels}}}$$

Vessel grouping indices.

### Intervessel contact fraction (*F*_C_) and intervessel contact length fraction (*F*_LC_)

The intervessel contact fraction (*F*_C_) is defined as the portion of the vessel wall in contact with other vessels (Wheeler et al., 2005; Hacke et al., 2006; Jansen et al., 2011). *F*_C_ values are calculated on transverse wood sections as the ratio of the sum of the intervessel contact perimeter to the sum of the total vessel perimeter. Assuming that vessel end walls are randomly arranged in wood, a single cross section will contain similar vessel contact fractions as measurements along the entire longitudinal plane of vessels.

(10) $$F_{\text{C}}=\frac{\sum L_{\text{VW}}}{\sum P_{\text{V}}}$$

Calculation of the vessel contact fraction *F*_C_. *L*_VW_, intervessel wall length; *P*_V_, vessel perimeter.

The intervessel contact length fraction (*F*_LC_) is the amount of vessel length that is in contact with other vessels. This parameter equals all the vessels in contact with other vessels.

(11) $$F_{\text{LC}}=\frac{L_{\text{C}}}{L_{\text{V}}}\;L_{\text{C}}=L_{\text{V}}\times (1-V_{S})\;F_{\text{LC}}=(1-V_{S})$$

Calculation of the intervessel contact length fraction *F*_LC_ based on *V*_S_. *L*_C_, intervessel contact length.

## Pit dimensions

Pits are openings in the secondary cell wall of conduits. All water transporting cells show bordered pits, while non-water conducting cells have usually non-bordered (i.e., simple) and less frequently bordered pits (Carlquist, 2007; Choat et al., 2008; Sano et al., 2011). The bordered pits of angiosperms can easily be distinguished from the torus-margo structure of gymnosperms. Quantification of pits frequently requires careful observations using a light microscope with a 100 × oil-immersion objective, as well as electron microscopy (TEM and SEM) for the observation of ultrastructural details. Care should be taken to distinguish different pit types between conduits, such as intervessel pits, vessel-fiber pits, and vessel-parenchyma pitting. The micromorphology, size, and arrangement of these pits and pit membranes may vary considerably between these pit types (Choat et al., 2008; Jansen et al., 2009).

### Pit surface area (*A*_PIT_) [μm^2^]

The pit surface area *A*_PIT_ is the area occupied by the pit border or the pit membrane area between conduits. For gymnosperms, the surface area of the torus and the margo can be measured separately (Hacke and Jansen, 2009). It is also useful to measure the area occupied by the pit aperture *A*_PA_, or to get additional information of the shape of the aperture by measuring the longest and shortest diameter of the pit aperture (*D*_PA long_, *D*_PA short_).

### Pit membrane diameter (D_PM_) [μm]

Measurements of the pit membrane diameter (*D*_PM_) can be calculated via the pit surface area or directly measured on the pits. In gymnosperms the ratio of the torus diameter (*D*_TO_), margo diameter (*D*_MA_), and the diameter of the aperture measured at its widest point (*D*_PA_) have been used to quantify the amount of torus overlap. This feature has been shown to be predictive of cavitation resistance, with species that are highly vulnerable to cavitation showing a small torus overlap compared to more resistant species (Sperry and Tyree, 1990; Hacke and Jansen, 2009; Delzon et al., 2010).

### Pit membrane thickness (T_PM_) [nm]

A surprisingly large variation exists in the pit membrane thickness of bordered pits in angiosperms, which vary from ca. 100 nm to >1000 nm in thickness (Jansen et al., 2009). These observations require the use of fresh material and TEM, because pit membranes shrink considerably in dried samples. Measurements can be made near the center of the pit membrane, at the thinnest part, or the thickest part of the membrane. Although these measurements are time-consuming, pit membrane thickness appears to be a good indication of pit membrane porosity and has been found to be closely correlated with cavitation resistance (Jansen et al., 2009; Lens et al., 2011; Plavcová et al., 2011).

### Pit chamber depth (*D*_PC_) [μm]

The intervessel pit chamber depth *D*_PC_ is measured as the distance from the “roof” of a pit border to the adjacent pit bordered. Dividing this value by two gives the pit chamber depth of a single pit border, which estimates the distance that the pit membrane can be pushed to become aspirated (i.e., pushed against the outer pit aperture).

### Intervessel pitfield fraction (*F*_PF_)

*F*_PF_ is the intervessel pitfield fraction or the ratio of intervessel surface area occupied by intervessel pits to total intervessel wall area. Measurements can be done with light microscopy or scanning electron microscopy. The use of SEM images is more accurate as the delimitation of a single pit is easier to determine using SEM than LM.

### Interconduit pit membrane area (*A*_P_) [mm^2^]

A model to estimate the total area of interconduit pit membranes (*A*_P_) was developed by Wheeler et al. (2005). Quantifying the amount of intervessel or intertracheid pit membrane area per average vessel or tracheid has been useful to test the rare-pit hypothesis, which states that vulnerability to cavitation scales negatively to the amount of *A*_P_, suggesting that the size of the largest pit membrane pore that triggers air-seeding depends on the amount of interconduit pitting (Hacke et al., 2006; Christman et al., 2009). A brief protocol for calculating *A*_P_ is given below (Equation 13).

### Intervessel pit fraction (*F*_P_)

Intervessel pit fraction *F*_P_ resembles the fraction of the total vessel wall area occupied by intervessel pits. To calculate *F*_P_ we first measure the contact fraction *F*_C_ (see Equation 10), i.e., the portion of the vessel wall that is in contact with other vessels. Then, the pit area per contact area or the pitfield fraction (*F*_PF_) is measured.

(12) $$F_{\text{P}}=F_{\text{C}}\times F_{\text{PF}}$$

The total area of intervessel pit membranes (*A*_P_) is derived from the pit fraction (*F*_P_) and the vessel surface area (*A*_V_).

(13) $$A_{\text{P}}=F_{\text{P}}\times A_{\text{V}}$$

(14) $$A_{\text{V}}=\pi \times D_{\text{H}}\times L_{\text{V}}$$

For rectangular tracheids, the tracheid surface area (*A*_T_) is calculated instead of *A*_V_ using the following formula:

(15) $$A_{\text{T}}=4\times D_{\text{H}}\times L_{\text{V}}$$

## The vulnerability and mesomorphy index

The vulnerability index (*VI*) after Carlquist (1977) is calculated using the vessel diameter (*D*, μm) and the vessels density (*V*_D_, mm^−2^) and provides a rough indication of the plant to withstand drought- or frost-induced cavitation. *VI* values below 1.0 suggest a high degree of xeromorphy, while values above 3.0 would characterize mesomorphy. A similar index called the mesomorphy index (*MI*) is calculated by multiplying the vulnerability index (*VI*) with the vessel element length (*L*_VE_) (Carlquist, 1977). MI values below 30.0 indicate true xeromorphy and values around 200 or higher suggest mesomorphy. Although both indices have been criticized (Lens et al., 2011), they rely on parameters that can easily be measured, including fossil wood samples.

$$VI=\frac{D}{V_{\text{D}}}$$

(16) $$MI=VI\times L_{\text{VE}}$$

Calculating the different ecological indices, *VI*—vulnerability index and *MI*—mesomorphy index after Carlquist (1977).

## Image analysis tools and hardware

Various image analysis packages and programs can be used such as WinCELL, ROXAS (Von Arx et al., 2012), Image Pro Plus (Media cybernetics, Silverspring, MA, USA), and ImageJ (Rasband, 1997–2004). We have good experience using a combination of two programs, the freeware ImageJ and the commercial software Image Pro Plus. ImageJ is available in several versions and can be used in combination with many plugins and macros (Table 3). A useful version of ImageJ that was developed by the McMaster Biophotonics Facility is downloadable at http://www.macbiophotonics.ca/imagej/. ImageJ also allows 3D reconstruction based on 2D sections and several stack operations (Table 3).

**Table 3:** **Overview of useful plugins for image analysis using ImageJ**.

**Plugin name**	**Operation**	**Function**
Cell counter	Plugins → Particle Analyses → Cell Counter	Manual counting of up to 3 different cell types in a single image (e.g., vessels, tracheids).
Multi measure	Plugins → ROI → Multi Measure	Manual measuring of multiple distances, lengths, polygons, etc., which can be burnt into your image.
Threshold	Image → Adjust → Threshold	Automatic selection of structures of interest based on their grey values.
Analyze particles	Analyze → Analyze Particles	Automatic counting of cells of a certain size or shape. A threshold for size and shape can be set prior to measuring.

A trial version of Image Pro Plus program can be downloaded at http://www.mediacy.com/index.aspx?page=IPP. The main advantage of Image Pro Plus in comparison to ImageJ is the option to select, deselect, delete, and add single objects of an image after automatic analysis. By automatically transforming the selected objects into a single vector, one may easily include non-detected objects or delete falsely identified ones. Image Pro Plus includes a larger number of simultaneously measurable characters than ImageJ, which may speed up the analyses. Several additional modules and options are available in Image Pro Plus, such as Image Recording options, which directly connect your camera to Image Pro Plus, 3D Reconstruction, Automatic Stitching, tracking of cells trough the Z-axis, etc.

It may also be useful to stitch several images of high resolution together. Optimal results are obtained when the images recorded show 1/3 overlap. There are various stitching tools freely available for ImageJ (e.g., http://fly.mpi-cbg.de/~preibisch/software.html).

Image analysis programs may not be very useful if the image quality is low. In such case, we do not recommend any automated analyses, but manual counting and measuring of conduit characters. The use of a graphic pad instead of a standard mouse could be useful to speed up the manual selection and measurement of features, especially the tracing of structures with poor contrast. We have good experience with a touch screen graphic pad WACOM CINTIQ 12 WX (Wacom Europe GmbH, Germany), which includes a pen and several pen leads.

Nevertheless, our experience is that “old-fashioned” techniques such as the use of a drawing tube attached to a light microscope in combination with a graticule can be at least equally accurate and sometimes even faster than any digital approach.

### Conflict of interest statement

The authors declare that the research was conducted in the absence of any commercial or financial relationships that could be construed as a potential conflict of interest.
